# Is There an Association Between Diet, Physical Activity and Depressive Symptoms in the Perinatal Period? An Analysis of the UPBEAT Cohort of Obese Pregnant Women

**DOI:** 10.1007/s10995-020-02933-3

**Published:** 2020-04-30

**Authors:** Claire A. Wilson, Paul Seed, Angela C. Flynn, Louise M. Howard, Emma Molyneaux, Julie Sigurdardottir, Lucilla Poston

**Affiliations:** 1 Section of Women’s Mental Health, Institute of Psychiatry, Psychology and Neuroscience, King’s College London and South London and Maudsley NHS Foundation Trust, PO31 King’s College London, De Crespigny Park, London, SE5 8AF UK; 2 Department of Women and Children’s Health, King’s College London, St Thomas’ Hospital, 10th floor North Wing, London, SE1 7EH UK

**Keywords:** Perinatal depression, Physical activity, Glycaemic load, Saturated fat

## Abstract

**Introduction:**

Depression is a common morbidity of the perinatal period (during pregnancy and up to one year postpartum). There is evidence for an association between diet and physical activity, and depression in the non-pregnant population but this association has been relatively less explored during the perinatal period; particularly poorly understood is the relationship between specific dietary components and depression. The aim of this study was to explore the association between glycaemic load, saturated fat intake and physical activity and depressive symptoms in a high-risk population of obese pregnant women.

**Methods:**

In a cohort of 1522 women participating in the UPBEAT trial, physical activity, glycaemic load and saturated fat intake were used as predictors of depressive symptoms measured using the Edinburgh Postnatal Depression Scale (EPDS). Measures taken in early pregnancy were used in linear and logistic regression models. Repeated measures at three points during pregnancy and at six months postpartum were utilised in multilevel mixed effects models. Multiple imputation was used to account for missing data.

**Results:**

Increased glycaemic load was associated with small increases in levels of depressive symptoms across the perinatal period (adjusted beta coefficient 0.01; 95% CI 0.01,0.02). There was no evidence for an association between reduced physical activity and increased saturated fat intake and increased levels of depressive symptoms.

**Conclusions:**

Glycaemic load may be a useful focus for interventions aiming to optimise the mental health of obese women in the perinatal period.

**Electronic supplementary material:**

The online version of this article (10.1007/s10995-020-02933-3) contains supplementary material, which is available to authorized users.

## Significance

*What is already known on this subject? *There is some evidence for an association between both diet and physical activity, and depression in the general and pregnant populations.

*What this study adds? *Increased glycaemic load in a cohort of obese pregnant women was associated with increased depressive symptoms measured from early pregnancy to six months postpartum. Greater understanding of this relationship between specific dietary components and depression in the perinatal period provides an insight into potential targets for treatment and intervention in this population.

## Introduction

A growing body of evidence supports a bidirectional relationship between mental health and nutrition in the non-pregnant population (Sarris et al. 2015). Much of the research has focussed on depression, which is a substantial contributor to burden of disease globally (Vigo et al. 2016). Observational studies suggest that a range of nutritional components may be associated with depression. These include relationships with specific nutrient deficiencies, the most frequently studied being vitamin D (Eyles et al. 2013) and omega-3 fatty acids (Mischoulon and Freeman 2013). Systematic reviews have explored relationships with dietary quality or patterns; the most consistent findings being that a ‘Mediterranean’ diet high in fruit, vegetables and whole grains is associated with a reduced risk of depression compared to a diet high in refined sugar and processed foods (Lassale et al. 2018). While it is not clear if this relationship is causal, studies investigating the impact of specific food groups on risk for depression may give some insights in to potential mechanisms underlying this relationship with dietary patterns. For example, positive associations have been observed between high intake of saturated fat and depression (Melo et al. 2019) and between high glycaemic index (GI) and glycaemic load (GL) diets in both observational and intervention studies (Salari-Moghaddam et al. 2019). Indeed a number of intervention studies have demonstrated that depression can be effectively treated either by correcting specific micronutrient deficiencies (Rucklidge and Kaplan 2013) or promoting a Mediterranean-style diet (Jacka et al. 2017). Systematic review level evidence also suggests that physical activity protects against the onset of and is an effective treatment for depression (Schuch and Stubbs 2019). These benefits are observed at a range of intensities and durations of physical activity (Schuch and Stubbs 2019).

There is also evidence supporting a bidirectional association between obesity and depression in the non-pregnant population (Mannan et al. 2016) but the relationship between diet, physical activity and depression has been less studied in obese populations than in those of healthy weight. However, there is some evidence that the relationship between diet, specifically glycaemic load, and depression may be moderated by body weight, based on observations of stronger evidence for associations between mood and glycaemic load in those with overweight or obesity than in those of healthy weight (Breymeyer et al. 2016). There are also a number of studies in which reductions in depressive symptoms have been associated with physical activity interventions of two to three months duration involving jogging (Irandoust and Taheri 2017) and group exercise (Hayward et al. 2000) in populations of obese women. Another population in which the impact of diet and physical activity on risk for depression has been relatively less explored is in women in the perinatal period (during pregnancy and up to one year postpartum), yet mental disorders are the commonest morbidity of the peripartum, with around 10% of women affected by depression during this period (Howard et al. 2014).

Regarding diet in the perinatal period, a systematic review of nine observational studies suggested cross-sectional associations between an unhealthy ‘Western’ diet (defined as high in refined sugar and processed foods) and antenatal depression but insufficient evidence to support an association with postnatal depression (Baskin et al. 2015). Limitations of the included studies highlighted in the review was that their designs allowed for only one measurement of diet and that diet is likely to vary throughout pregnancy. Indeed, depressive symptoms also tend to fluctuate during the perinatal period (Lee et al. 2007). A more recent review found some evidence for an association between a ‘healthy’ diet of fruit, vegetables, fish and whole foods, and a reduced risk of postnatal depression (Silva et al. 2019). However, another systematic review remarked that many of the perinatal nutrition interventions, most of which were of vitamin D or fish oils, neglected to measure postnatal depression (Gould et al. 2017).

In regard to physical activity, the most recent reviews including observational studies and intervention studies with exercise-based interventions for the prevention and treatment of perinatal depression have been inconclusive and generally report small effect sizes (Carter et al. 2019; Saligheh et al. 2017); they are also usually postnatal interventions, as historically there has been less focus on optimising the mental health of women during pregnancy (Shivakumar et al. 2011).

A growing body of evidence suggests a strong relationship between obesity and depression not only in the non-pregnant population but also in pregnancy (Molyneaux et al. 2014). However, there is limited research addressing the relationship between diet, physical activity, and depression in this group of high risk women. Thus the aim of our study was to investigate the relationship between physical activity and the specific dietary components of glycaemic load and saturated fat intake and depressive symptoms in a cohort of obese pregnant women who had participated in UPBEAT (UK Pregnancies Better Eating and Activity Trial). UPBEAT was a multicentre randomised controlled trial of a behavioural intervention focussed on increasing physical activity and improving diet in obese pregnant women (Briley et al. 2014). Measures of diet, physical activity and depressive symptoms were taken at three visits during pregnancy and at six months postpartum. The intervention recommended consumption of a diet with a low glycaemic load and low intake of saturated fat together with increased physical activity. As previously reported, the intervention did not reduce the incidence of the primary outcomes of gestational diabetes (GDM) and large-for-gestational-age infants but did result in modest reductions in glycaemic load and saturated fat intake and increases in physical activity (Poston et al. 2015). We have also previously reported that the intervention did not affect levels of depressive symptoms (Molyneaux et al. 2018). In light of these findings, in this study we sought to examine the association between diet and physical activity and depressive symptoms independently of the intervention. We hypothesised that there would be an association between reduced physical activity, increased glycaemic load and increased saturated fat intake and increased levels of depressive symptoms.

## Methods

This is a pre-planned secondary analysis of the UPBEAT study data.

### Sample

**Fig. 1:**
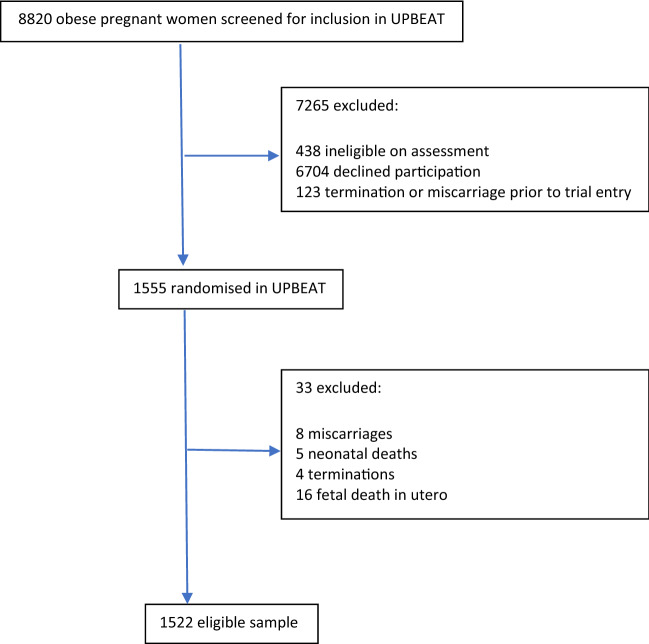
Participant flow

The UPBEAT study recruited 1555 women from eight UK NHS hospital trusts between March 2009 and June 2014 at 15 to 18 weeks’ gestation who were greater than 16 years of age with body mass index (BMI) ≥ 30 kg/m^2^ and a singleton pregnancy. Exclusion criteria were lack of informed consent, current use of metformin and a range of pre-pregnancy medical conditions (Briley et al. 2014). 33 women were excluded from our analysis due to either miscarriage, termination of the pregnancy, fetal death in utero or neonatal death, leaving a sample of 1522 women (Fig. 1). All women gave informed consent prior to their inclusion in the study.

Women randomised to the intervention received up to eight weekly one hour sessions with a health trainer, a pedometer, DVD, handbook and log book: all aimed at supporting dietary and physical activity goal setting. The primary aim was to improve glucose tolerance, with the primary outcome being a reduction in the incidence of GDM and large for gestational age infants. The dietary component of the intervention focused on reducing glycaemic load and saturated fat intake. To reduce glycaemic load, participants were encouraged to swap foods and beverages with a high glycaemic index for those with a lower glycaemic index and to reduce the consumption of sugar-sweetened beverages, including fruit juice. To reduce saturated fat, participants were encouraged to use low fat dairy products and exchange fatty meats and meat products with leaner meat and fish. The physical activity component focused on walking at a moderate intensity, with additional options for participants already engaged in some physical activity. The control group received standard antenatal care. NHS research ethics committee approval was obtained for all study centres (UK integrated research application system reference: 09/H0802/5).

## Measures

### Predictors

#### Diet

Diet was assessed in all participants using a food frequency questionnaire (FFQ) adapted from the UK arm of the European Prospective Investigation into Cancer Study (EPIC) (Briley et al. 2014). The questionnaire was a shortened version of that used in EPIC and focused on evaluating food groups pertinent to the UPBEAT dietary intervention. Questions relating to carbohydrate sources were detailed to differentiate low from high glycaemic index varieties. Questions relating to dietary fat sources distinguished high from low saturated fat varieties.

The questionnaire was administered (1) at study baseline (15 to 18 weeks’ gestation), (2) at follow-up during pregnancy at 27 to 28 weeks’ gestation, (3) at 34 to 36 weeks’ gestation and (4) at the six months postpartum follow-up visit. Glycaemic load (grams per day) and saturated fat intake as a percentage of energy per day (gross calorific intake) were calculated. Glycaemic load is the amount of carbohydrate multiplied by its glycaemic index, glycaemic index being how much carbohydrate will raise a person’s blood glucose.

### Physical Activity

Physical activity was measured at the three visits during pregnancy: 15 to 18, 27 to 28 and 34 to 36 weeks’ gestation and at six months postpartum using the validated International Physical Activity Questionnaire (IPAQ). Physical activity was expressed as minutes per week and calculated as metabolic equivalents (METs), which is the ratio of energy expenditure for an activity to energy expenditure at rest, then using the formula: (8.0xvigorous activity) + (4.0xmoderate activity) + (3.3xlight activity) (Briley et al., 2014). MET was expressed as a square root as the data were not normally distributed.

### Outcome

#### Depressive Symptoms

Symptoms of depression were measured at 15 to 18, 27 to 28 and 34 to 36 weeks’ gestation and at six months postpartum using the validated Edinburgh Postnatal Depression Scale (EPDS) (Cox et al. 1987). Scores were expressed as both linear (out of a maximum of 30) and binary outcomes (≥ 13 or < 13 as a validated cut-off score for high levels of depressive symptoms) (Murray and Cox 1990).

### Covariates

There are a number of potential confounders associated with the predictors of diet and physical activity and outcome of depressive symptoms in this study. Effect estimates were adjusted for age and also these confounders.

1. BMI (kg/m^2^): calculated from height and weight, measured at baseline 15 to 18 weeks’ gestation.
2. Deprivation: index of multiple deprivation (IMD) was assigned based on postcode and expressed as quintiles of the English population; adjusted values were used for Scottish addresses (Department for Communities and Local Government 2015; Scottish Government 2009).
3. Ethnicity: participants reported their main ethnicity which was categorised as Asian, Black, White or Other.
4. Randomisation to the intervention: intervention versus control group.

### Statistical Analysis

For the purposes of this study the trial population was treated as a cohort as we were interested in the association between diet and physical activity and depressive symptoms in the population as a whole, not the effect of the intervention as has been studied previously. The regress and logistic commands in Stata version 15 (StataCorp, College Station, Texas, USA) were used to construct multivariable linear and logistic regression models for baseline (15 to 18 weeks gestation) linear and binary outcomes respectively. Baseline physical activity (MET), glycaemic load and saturated fat were considered as predictors in separate models. The covariates of age, BMI, deprivation, ethnicity and randomisation to the intervention were also included in adjusted analyses.

The mixed and meqrlogit Stata commands were then used to construct multilevel mixed effects models for linear and binary outcomes respectively, making use of the repeated measures of EPDS, physical activity, glycaemic load and saturated fat through the peripartum at the four study visits. Physical activity, glycaemic load and saturated fat were again considered as predictors in separate models. A mean score for each predictor in each participant was used as an indicator of the mean across the peripartum for that participant, in addition to the repeated measures of the predictor throughout the peripartum for each participant, in order to assess whether or not changes in the predictor were associated with changes in the outcome. For these mixed effects models, an independent covariance matrix was used and the same set of covariates used as in baseline analyses, in addition to the covariate of time (study visit). Evidence of an association was interpreted as the 95% confidence interval for the effect estimate not crossing the null value (for the beta coefficient this is zero and for odds ratio is one).

Multiple imputation by chained equations was implemented to handle missing data (White et al. 2011). Data were imputed separately for continuous and binary outcomes (EPDS scores). 40 imputations were used to reflect the proportion of the sample without complete covariate data and without at least one measure of each predictor and the outcome (Bodner 2008). All analysis variables were included in the imputation models. Estimates were obtained by pooling results using Rubin’s rules (Rubin 1987). Imputed data were used for the primary analysis and a complete case analysis was performed as a sensitivity analysis.

## Results

The characteristics of the sample of 1522 women, including levels of missingness for each variable due to incomplete measures and differences between complete (with a baseline measure of depressive symptoms, glycaemic load, saturated fat and physical activity and complete covariate data) and incomplete cases are presented in Table 1. Mean age of the 1522 women was 30.5 years and median BMI was 34.6. Over 75% of the sample were in the two most deprived deprivation quintiles. 63% of the sample were of White ethnicity. 11% scored ≥ 13 on the EPDS at baseline, indicating ‘caseness’ for depression.Complete cases were more of White and Asian ethnicity than Black ethnicity. They were also less physically active.

**Table 1:** Characteristics of the sample

	Total sample N = 1522	Complete cases N = 945*	Incomplete cases N = 577	Significance (complete v incomplete cases)**
Age (years)				Difference (95% CI)
Mean (SD)	30.5 (5.5)	30.4 (5.5)	30.4 (5.5)	− 0.2 (− 0.4,0.7)
Missing n (%)	1 (0.1)			p = 0.60
BMI (kg/m^2^)				Z: 0.22
Median (IQR)	34.6 (32.3–38.1)	34.5 (32.3–38.0)	34.7 (32.2–38.4)	p = 0.83
Missing n (%)	13 (0.9)			
Index of multiple deprivation quintiles				Chi square: 5.45
n (%)				p = 0.24
1 (least deprived)	62 (4.1)	40 (4.2)	22 (3.9)	
2	100 (6.6)	60 (6.4)	40 (7.0)	
3	173 (11.4)	119 (12.6)	54 (9.5)	
4	523 (34.4)	311 (32.9)	212 (37.2)	
5 (most deprived)	657 (43.2)	415 (43.9)	242 (42.5)	
Missing	7 (0.5)			
Ethnicity n(%)				Chi square: 19.25
Asian	91 (6)	62 (6.6)	29 (5.0)	p < 0.01
Black	391 (25.7)	207 (21.9)	184 (31.9)	
White	955 (62.8)	622 (65.8)	333 (57.8)	
Other	84 (5.5)	54 (5.7)	30 (5.2)	
Missing	1 (0.1)			
Intervention n(%)				Chi square: 0.74
Yes	768 (50.5)	485 (51.3)	283 (49.1)	p = 0.39
No	754 (49.5)	460 (48.7)	294 (50.9)	
Missing	0 (0)			
Baseline EPDS ≥ 13 n (%)				Chi square: 2.64
≥ 13	163 (10.7)	107 (11.3)	56 (14.6)	p = 0.10
< 13	1167 (76.7)	838 (88.7)	329 (85.4)	
Missing	192 (12.6)			
Baseline EPDS depression score (/30)				Z: 0.87
Median (IQR)	6 (3–10)	6 (3–10)	6 (3–10)	p = 0.38
Missing n (%)	192 (12.6)			
Baseline MET (mins/week)				Difference (95% CI)
Median (IQR)	3383.3 (4960.2)	1434 (693–3744)	1386 (522–3177)	− 16.3 (-63.6, − 0.01)
Square root transformed mean (SD)	47.7 (33.3)	48.9 (33.8)	44.8 (31.9)	p = 0.04
Missing n (%)	191 (12.5)			
Glycaemic load (grams/day)				Difference (95% CI)
Mean (SD) baseline	136.7 (52.6)	136.5 (51.4)	138.1 (59.0)	1.7 (-6.9,10.2)
Missing n (%)	403 (26.5)			p = 0.70
Saturated fat (% energy)				Difference (95% CI)
Mean (SD) baseline	12.6 (3.0)	12.6 (3.0)	12.7 (2.9)	0.1 (− 0.3,0.6)
Missing n (%)	403 (26.5)			p = 0.56

^*^ Complete baseline EPDS, glycaemic load, saturated fat and physical activity (MET) and complete covariate data

^**^Chi square for categorical variables, t test for parametric continuous variables and Mann Whitney for non-parametric continuous variables

Table 2 displays the results of imputed unadjusted and adjusted linear and logistic regression models at baseline (15 to 18 weeks gestation). There was little evidence of an association between physical activity or saturated fat intake at baseline and depressive symptoms as both continuous and binary outcomes. However, there was evidence of a potential association with glycaemic load on linear (unadjusted beta 0.014; 95% CI 0.008,0.019) and logistic analyses (unadjusted OR 1.008; 95% CI 1.004,1.011). This effect was only slightly attenuated by adjustment for BMI, deprivation, ethnicity and participation in the intervention (adjusted beta 0.012; 95% CI 0.007,0.018 and adjusted OR 1.007; 95% CI 1.004,1.010). This translates to an increase in EPDS score of 0.012 (out of a total of 30) for every one unit increase in glycaemic load or the odds of depression (EPDS score ≥ 13) increasing by 1.007 times for each one unit increase in glycaemic load. Results of complete case analyses yielded a similar pattern of results and are presented in electronic supplementary material.

**Table 2:** Associations of physical activity, glycaemic load and saturated fat with EPDS depressive symptoms at baseline (15 to 18 weeks gestation) N = 1522

Physical activity (square root of MET)					Glycaemic load (grams/day)					Saturated fat (% energy)				
Linear regression														
	Unadjusted		Adjusted*			Unadjusted		Adjusted*			Unadjusted		Adjusted*	
	Beta (95% CI)	p value	Beta (95% CI)	p value		Beta (95% CI)	p value	Beta (95% CI)	p value		Beta (95% CI)	p value	Beta (95% CI)	p value
EPDS (/30)	-0.008 (-0.016, 0.001)	0.08	-0.005 (-0.013, 0.004)	0.26	EPDS (/30)	0.014 (0.008, 0.019)	< 0.01	0.012 (0.007, 0.018)	< 0.01	EPDS (/30)	-0.038 (-0.138, 0.062)	0.46	0.034 (-0.106,0.099)	0.94
Logistic regression														
	Unadjusted		Adjusted*			Unadjusted		Adjusted*			Unadjusted		Adjusted*	
EPDS ≥ 13 Mean (SD) of physical activity	OR (95% CI)	p value	OR (95% CI)	p value	EPDS ≥ 13 Mean (SD) of glycaemic load	OR (95% CI)	p value	OR (95% CI)	p value	EPDS ≥ 13 Mean (SD) of saturated fat	OR (95% CI)	p value	OR (95% CI)	p value
< 13 *ref* 47.9 (33.4)	0.999 (0.993,1.004)	0.67	1.001 (0.995,1.006)	0.81	< 13 *ref* 130.5 (50.5)	1.008 (1.004,1.011)	< 0.01	1.007 (1.004,1.010)	< 0.01	< 13 *ref* 12.6 (2.9)	0.969 (0.913, 1.028)	0.29	0.988 (0.928,1.050)	0.69
** ≥ 13** 46.7 (32.7)					** ≥ 13** 154.7 (59.1)					** ≥ 13** 12.3 (3.1)				

Using imputed data *adjusted for BMI, deprivation quintile, ethnicity, age and participation in the intervention

Table 3 displays the results of unadjusted and adjusted multilevel mixed effects (within and between participants) linear and logistic regression models. Making use of the repeated measures of the variables throughout the peripartum had little impact on the results. There remained little evidence of an association between physical activity or saturated fat intake and depressive symptoms. The effect of glycaemic load as a repeated measure throughout the peripartum and the mean of these repeated measures remained similar to that at baseline. Variance component estimates for the multilevel mixed effects models and their standard errors are also displayed in Table 3. The sum of the variance component estimates reflects the variability in the outcome that remains after controlling for variables included in the models. Variance estimates between participants and within participants are presented. These were broadly similar across the three sets of predictors and the variance within participants was greater than that between participants.

**Table 3:** Multilevel mixed effects model of associations of physical activity, glycaemic load and saturated fat with EPDS depressive symptoms across the peripartum N = 1522

	Physical activity (square root of MET)				Glycaemic load (grams/day)				Saturated fat (% energy)			
Linear regression												
	Unadjusted*		Adjusted**		Unadjusted*		Adjusted**		Unadjusted*		Adjusted**	
	Beta (95% CI)	p value	Beta (95% CI)	p value	Beta (95% CI)	p value	Beta (95% CI)	p value	Beta (95% CI)	p value	Beta (95% CI)	p value
Participant mean	-0.002 (-0.011,0.007)	0.68	0.001 (-0.009,0.010)	0.90	0.009 (0.003,0.016)	< 0.01	0.008 (0.002,0.015)	0.01	-0.024 (-0.129,0.082)	0.66	0.021 (-0.090,0.132)	0.71
Repeated measure of predictor	-0.005 (-0.010,0.001)	0.08	-0.005 (-0.011,0.001)	0.08	0.010 (0.005,0.014)	< 0.01	0.010 (0.005,0.015)	< 0.01	0.027 (-0.048,0.102)	0.47	0.026 (-0.048,0.101)	0.49
Logistic regression												
	Unadjusted*		Adjusted**		Unadjusted*		Adjusted**		Unadjusted*		Adjusted**	
	OR (95% CI)	p value	OR (95% CI)	p value	OR (95% CI)	p value	OR (95% CI)	p value	OR (95% CI)	p value	OR (95% CI)	p value
Participant mean	1.001 (0.993,1.009)	0.73	1.004 (0.996,1.012)	0.38	1.007 (1.001,1.012)	0.02	1.006 (1.001,1.012)	0.02	0.958 (0.881,1.041)	0.31	0.985 (0.903,1.073)	0.72
Repeated measure of predictor	0.997 (0.992,1.002)	0.25	0.997 (0.992,1.002)	0.24	1.008 (1.004,1.012)	< 0.01	1.008 (1.004,1.012)	< 0.01	1.009 (0.953,1.070)	0.75	1.009 (0.952,1.069)	0.77
	Random effects on linear analyses				Random effects on linear analyses				Random effects on linear analyses			
	Variance	SE	Variance	SE	Variance	SE	Variance	SE	Variance	SE	Variance	SE
Between participants	2.536	0.102	2.463	0.102	2.132	0.149	2.048	0.150	2.569	0.159	2.544	0.086
Within participants	3.908	0.058	3.910	0.058	3.871	0.058	3.871	0.058	3.930	0.059	3.932	0.058

Using imputed data *adjusted for time of visit only. **adjusted for BMI, deprivation quintile, ethnicity, age, participation in the intervention and time of visit

## Discussion

### Main Findings and Potential Mechanisms

This analysis in a cohort of obese pregnant women provides evidence for an association between increased glycaemic load and increased levels of depressive symptoms across the peripartum. We found insufficient evidence for an association between reduced physical activity and increased saturated fat intake and increased levels of depressive symptoms. However, the effect sizes are relatively small.

An association between glycaemic load and depression but not saturated fat intake is perhaps surprising given that saturated fat and sugar are common constituents of much of the ‘unhealthy’ dietary patterns reported in the diet and mood literature in the non-pregnant population, in which some positive associations have been observed between this unhealthy diet and increased risk of depression (Lassale et al. 2018). Despite this, a recent review found inconclusive evidence for such a relationship in the perinatal population (Silva et al. 2019). Clearly factors other than nutrition could influence the development of perinatal depression and we discuss these challenges to causal inference in strengths and limitations below. However, a number of possible mechanisms may underlie an association between diet and depression. Some essential nutrients are required for the synthesis of key neurotransmitters implicated in the pathophysiology of depression (Leung and Kaplan 2009). Moreover, inflammation is a pathology common to both poor diet and depression and an association has been observed between a pro-inflammatory diet and depression, particularly in women (Wang et al. 2018). However, the biological mechanisms of any relationship between diet and depression in the perinatal period require further investigation.

Specifically in relation to the association between depression and glycaemic load, as outlined previously glycaemic load is the amount of carbohydrate multiplied by its glycaemic index. Some have hypothesised that increased carbohydrate intake leads to enhanced delivery to the brain of tryptophan: a precursor of the neurotransmitter serotonin, which is implicated in the pathophysiology of depression. This is particularly relevant when considering mood in the perinatal period, as a decline in tryptophan has been suggested to occur in the early postnatal period, associated with a fall in plasma insulin following delivery of the placenta; insulin facilitates the transport of tryptophan. Thus our findings are surprising as this hypothesis has led to suggestions that a high carbohydrate diet may be a potential preventative strategy against the development of postnatal depression (Chen et al. 2006). However, one study in the non-pregnant population which found an association between high glycaemic load and higher depression scores hypothesised that hunger and fluctuations in blood sugar associated with higher glycaemic load was what underpinned this association (Cheatham et al. 2009). Furthermore, in an obese cohort of women with major depressive disorder, greater intake of total sugars was associated with increased levels of depressive symptoms (Appelhans et al. 2012).

The lack of an association between physical activity and perinatal depressive symptoms is consistent with much of the literature conducted in the perinatal period; studies that report positive associations often have small effect sizes in both observational and intervention studies (Carter et al. 2019; Saligheh et al. 2017; Shivakumar et al. 2011). However, evidence from non-pregnant populations has shown benefits associated with physical activity (Schuch and Stubbs 2019), although physical activity interventions in the general population may involve a broader range of activities than in the perinatal population; postnatal interventions are often focussed on vigorous walking so may be less intensive (Saligheh et al. 2017). In the UPBEAT cohort used for our study, women randomised to the intervention were encouraged to increase their walking with a pedometer but they were also given a DVD of an exercise regime safe for pregnancy (Briley et al. 2014). There are also a number of moderators of these potential benefits which have been noted in the general population (Schuch and Stubbs 2019); the role of both obesity and pregnancy as effect modifiers requires further exploration. Finally, we measured physical activity using MET derived from self-report; a large degree of heterogeneity in physical activity measurement has been noted in the perinatal literature (Nakamura et al. 2019) and may account for some of the variation in study results. More objective measures of physical activity may reduce measurement bias. Further limitations and strengths of our study are discussed below.

## Strengths and Limitations

This study explores the association between glycaemic load, saturated fat intake and physical activity and depressive symptoms in a cohort of obese pregnant women. A unique strength of this analysis is the availability of repeated measures of the exposures and outcome across the peripartum; much of the literature on diet and perinatal depression considers one point in the peripartum (Baskin et al. 2015; Silva et al. 2019) and on physical activity focusses mainly on the postpartum (Shivakumar et al. 2011). We were also able to focus on specific dietary components that were the targets of the intervention and had plausible mechanisms for an association with depression. We chose to analyse these as independent exposures due to significant collinearity between the saturated fat and glycaemic load variables and because we hypothesised that there may be potentially different mechanisms underpinning the relationships between depression and the exposures of glycaemic load, saturated fat and physical activity. However, we would have liked to explore other potential candidates such as free sugars, which were not measured during the intervention but which may exert differing effects on mood via a more rapid increase in blood glucose than that measured by glycaemic load.

While the cohort represents a particularly high risk group of women, the results may not be generalisable to other women with BMIs outside the obese range (< 30 kg/m^2^). Likewise while our diverse sample included a range of sociodemographic covariates, future research may usefully explore in larger samples the potential variations in our results between ethnic and socioeconomic groups. There may be different cultural values relating to diet and physical activity between ethnic groups and there is some evidence that socioeconomic factors may influence the relationships between diet, physical activity and depression in obese non-pregnant populations (Beydoun and Wang 2010). Other limitations are those that impede causal inference, including unmeasured confounding from variables not included in our analysis and reverse causality. Moreover, the inherent bias in self-reporting of dietary intake in FFQs is well documented (Shim et al. 2014). Finally it is important to remember that the EPDS is a screening tool to measure symptoms of depression. High scores only indicate possible ‘caseness’/high risk for depression; they do not equate to a diagnosis of depression.

### Conclusions for Practice

Despite these limitations, this analysis provides new insights into potential targets for optimising the mental health of obese women during the perinatal period. Previous evaluations of lifestyle interventions in overweight or obese pregnant women, including this UPBEAT trial as discussed in the introduction, did not find improvements in mental health from participation in the intervention (Altazan et al. 2019; Molyneaux et al. 2018). Yet other interventions in this population, including those focussing on advice about healthy diet, have been associated with positive impacts on mental health (Bogaerts et al. 2013). However, this analysis provides new understanding of the specific dietary components that may or may not be effective in the prevention and treatment of depression in this high-risk population. Development of interventions should consider those factors which may influence adherence, including sociocultural and psychological factors (Hamilton et al. 2018), although there is no evidence from a previous analysis of our cohort that depression specifically was associated with reduced adherence (Molyneaux et al. 2018). A focus on specific dietary components known to positively impact maternal mental health during the perinatal period may also have intergenerational effects (Jacka et al. 2013), establishing positive developmental trajectories in offspring and optimising the mental health of not only women but also their children.

## Electronic supplementary material

Below is the link to the electronic supplementary material.

Supplementary file1 (DOCX 25 kb)
