# Supplementary material for: Is There an Association Between Diet, Physical Activity and Depressive Symptoms in the Perinatal Period? An Analysis of the UPBEAT Cohort of Obese Pregnant Women
Source: Matern Child Health J. 2020 Apr 30;24(12):1482–93. doi: 10.1007/s10995-020-02933-3 (PMC7677286; doi:10.1007/s10995-020-02933-3)
Supplement: Supplementary file 1 — Supplementary file1 (DOCX 25 kb) [file 10995_2020_2933_MOESM1_ESM.docx]

Maternal and Child Health Journal

Is there an association between diet, physical activity and depressive symptoms in the perinatal period? An analysis of the UPBEAT cohort of obese pregnant women

*Dr Claire A Wilson^1^

Mr Paul Seed^2^

Dr Angela Flynn^2^

Prof Louise M Howard^1^

Dr Emma Molyneaux^1^

Mrs Julie Sigurdardottir^2^

Prof Lucilla Poston^2^

^1^Section of Women’s Mental Health, Institute of Psychiatry, Psychology and Neuroscience, King’s College London and South London and Maudsley NHS Foundation Trust, PO31 King’s College London, De Crespigny Park, London, SE5 8AF, UK

^2^ Department of Women and Children’s Health, King’s College London, 10^th^ floor North Wing, St Thomas’ Hospital, London, SE1 7EH, UK

*Corresponding author e-mail address: claire.wilson@kcl.ac.uk. Telephone: 07729 324100

*Complete case analyses of associations of physical activity, glycaemic load and saturated fat with EPDS depressive symptoms at baseline (15^+0^ to 18^+6^ weeks gestation)*

| **Physical activity (square root of MET)**  **N=1131** | | | | | **Glycaemic load (grams/day)**  **N=950** | | | | | **Saturated fat (% energy)**  **N=950** | | | | |
| --- | --- | --- | --- | --- | --- | --- | --- | --- | --- | --- | --- | --- | --- | --- |
| **Linear regression** | | | | | | | | | | | | | | |
|  | Unadjusted | | Adjusted* | |  | Unadjusted | | Adjusted* | |  | Unadjusted | | Adjusted* | |
|  | Beta (95% CI) | p value | Beta (95% CI) | p value |  | Beta (95% CI) | p value | Beta (95% CI) | p value |  | Beta (95% CI) | p value | Beta (95% CI) | p value |
| EPDS (/30) | -0.009  (-0.017,  -0.0005) | 0.04 | -0.007  (-0.015,  0.002) | 0.12 | EPDS (/30) | 0.013  (0.008,  0.019) | <0.01 | 0.011  (0.005,  0.017) | <0.01 | EPDS (/30) | -0.058  (-0.157,  0.042) | 0.25 | -0.043  (-0.145,0.058) | 0.40 |
| **Logistic regression** | | | | | | | | | | | | | | |
|  | Unadjusted | | Adjusted* | |  | Unadjusted | | Adjusted* | |  | Unadjusted | | Adjusted* | |
| EPDS ≥13  Mean  (SD) | OR (95% CI) | p value | OR (95% CI) | p value | EPDS ≥13  Mean  (SD) | OR (95% CI) | p value | OR  (95% CI) | p value | EPDS ≥13  Mean  (SD) | OR (95% CI) | p value | OR (95% CI) | p value |
| <13 *ref*  48 (33.6) | 0.999  (0.994,1.004) | 0.72 | 1.001  (0.995,1.006) | 0.77 | <13 *ref*  133.9  (50.0) | 1.008  (1.004,1.011) | <0.01 | 1.007  (1.004,1.010) | <0.01 | <13 *ref*  12.6  (2.9) | 0.964  (0.901,1.032) | 0.29 | 0.977  (0.910,1.048) | 0.51 |
| ≥13  46.9 (32.8) |  |  |  |  | ≥13  158.7  (58.1) |  |  |  |  | ≥13  12.3  (3.1) |  |  |  |  |

*adjusted for BMI, deprivation quintile, ethnicity, age and participation in the intervention.
